# Subtype-Specific Prevalence of Hepatitis C Virus NS5A Resistance Associated Substitutions in Mainland China

**DOI:** 10.3389/fmicb.2019.00535

**Published:** 2019-03-19

**Authors:** Jie Lu, Yupeng Feng, Lichang Chen, Zhengyu Zeng, Xianliang Liu, Wei Cai, Hui Wang, Xiaolei Guo, Huijuan Zhou, Wanyin Tao, Qing Xie

**Affiliations:** ^1^Department of Infectious Diseases, Ruijin Hospital, Shanghai Jiao Tong University School of Medicine, Shanghai, China; ^2^Guangzhou Kingmed Center for Clinical Laboratory Co., Ltd., Guangzhou, China; ^3^Hefei National Laboratory for Physical Sciences at Microscale, CAS Key Laboratory of Innate Immunity and Chronic Disease, School of Life Sciences, University of Science and Technology of China, Hefei, China

**Keywords:** HCV, NS5A, direct-acting antivirals, resistance associated substitutions, prevalence

## Abstract

Resistance associated substitutions (RASs) can reduce the efficacy of direct-acting antiviral agents (DAAs) targeting hepatitis C virus (HCV) and lead to treatment failure. Clinical data of HCV NS5A RASs prevalence are limited in China and need to be investigated. A total of 878 unique patient samples with different genotypes (GT) (1b: *n* = 489, 2a: *n* = 203, 3a: *n* = 60, 3b: *n* = 78, 6a: *n* = 48) were collected from around mainland China by KingMed Laboratory and analyzed for NS5A RASs distribution by Sanger sequencing. Phylogeographic analyses based on NS5A domain 1 sequences indicated circulation of both locally and nationally epidemic strains. Relatively high frequency of Y93H (14.1%) was only detected in GT1b but not in other subtypes. High frequency of L31M was found in both GT2a (95.6%) and GT3b (98.7%) sequences. Due to the overlapping incidence of A30K, 96% of GT3b isolates had NS5A RASs combination A30K + L31M, which confers high levels of resistance to most NS5A inhibitors. No RASs were detected in GT6a strains. Meanwhile, baseline NS5A RASs fingerprints were also evaluated in 185 DAA treatment-naive GT1b patients with next generation sequencing method. Patients presenting with Y93H had statistically higher entropy of HCV NS5A sequences. Taken together, subtype-specific distribution patterns of NS5A RASs were observed. GT1b patients with higher HCV complexity tend to have a greater chance of Y93H presence, while GT3b patients are naturally resistant to current NS5A inhibitors and their treatment may pose a challenge to real-world DAA application.

## Introduction

Hepatitis C virus infection is a global public health problem. HCV chronically infects about 3% of the world’s population, many of whom will develop liver cirrhosis and hepatocellular carcinoma (Mohd Hanafiah et al., 2013).

Hepatitis C virus has a high degree of genetic heterogeneity. It can be classified into seven GTs and numerous subtypes (Simmonds et al., 2005). In China, GT1b is estimated to account for more than 50% of total HCV infections (Chen Y. et al., 2017). GT3 is the next most prevalent globally and has increased dramatically in China (Messina et al., 2015; Lu et al., 2017). Meanwhile, HCV exists in host as quasispecies, which is defined as genetically distinct, but closely related viral populations (Martell et al., 1992). Quasispecies may play a role in disease progression and treatment response (Perales, 2018).

Direct-acting antiviral agents, targeting primarily the HCV NS3/4A protease, NS5A protein or NS5B polymerase, have replaced pegylated-interferon based therapy due to generally high sustained virological response (SVR) rate and good tolerance (Soriano et al., 2017). Among these DAAs, NS5A inhibitors are indispensable in most of the approved HCV antiviral regimens. Currently approved NS5A inhibitors include daclatasvir, ledipasvir, ombitasvir, elbasvir, velpatasvir, pibrentasvir, and ruzasvir (European Association for the Study of the Liver. Electronic address: easloffice@easloffice.eu, 2017; Soriano et al., 2017).

Resistance associated substitutions may result in decreased antiviral efficacy of the HCV DAAs and lead to treatment failure. The NS5B-substitution S282T, the only RAS with demonstrated *in-vitro* resistance to potent NS5B inhibitor sofosbuvir, was rarely seen at baseline and has been observed only in few patients at treatment failure (Svarovskaia et al., 2014; Xu et al., 2017). The majority of NS3 protease-resistant variants are present at low frequencies before DAA treatment except Q80K, which was frequently found in GT1a sequences but rarely seen in GT1b sequences (Sarrazin et al., 2015). In contrast, NS5A RASs are more prevalent in both DAA-naïve and DAA-experienced patients (Dietz et al., 2017). It is reported that patients with baseline NS5A RASs L31M/V and/or Y93H achieved much lower SVR rates than those without RASs (Karino et al., 2013). NS5A mutations at baseline influence the efficacy of ledipasvir / sofosbuvir regimen in GT1-infected patients (Zeuzem et al., 2017). Thus, the NS5A RASs distribution pattern becomes the focus of this study.

Available RASs prevalence data, mainly from DAA treatment-pioneer countries, showed NS5A RASs were detected at varied frequencies between GTs across geographic regions. RASs analyses based on 2761 sequences retrieved from the Los Alamos HCV database^1^ showed 6.1% of GT1b and 0.5% of GT1a sequences harbored L31M. As for M28V, 2.3% of GT1a and none of GT1b isolates harbored this substitution (Bagaglio et al., 2016). Data from 35 phase 1–3 studies in 22 countries showed the overall prevalence of baseline NS5A RASs was slightly higher in patients infected with GT1b (17.6%) than in those infected with GT1a (13%). Y93H was detected in 10.6% of GT1b patients and none in GT1a patients (Zeuzem et al., 2017). As for GT2, analyses based on 5 daclatasvir-containing clinical trials showed the most prevalent NS5A polymorphism was L31M, which was detected in 88% of GT2a, 59% of GT2b and 10% of GT2c isolates (Zhou et al., 2016). Global epidemiology of GT3 RASs showed NS5A A30K and L31M was detected more frequently in GT3b, 3g and 3k, while Y93H was only detected in GT3a (Welzel et al., 2017). Limited results of GT6 NS5A polymorphism did not reveal significant distribution of RASs (Welzel et al., 2017).

A few studies regarding RASs distribution in China have been published (Wang et al., 2015; Zhang et al., 2016; Chen Z.W. et al., 2017; Li et al., 2017; Wei L. et al., 2018). However, currently available data mainly focus on GT1b patients and are limited by the sample size. Therefore, the aim of this study is to explore the specific pattern of NS5A RASs distribution in general Chinese population and to clarify its impact on DAAs selection. HCV RNA-positive serum samples were collected across China and a nation-wide NS5A RASs prevalence investigation was performed. The subtype-specific NS5A genetic diversity and phylogenetic relationship of these NS5A sequences were analyzed. Due to the heterogenous distribution of a clinically important NS5A RAS, Y93H, in GT1b population, we then investigated its presence by nest-generation sequencing in a validation set of DAA treatment-naïve patients. The results presented here showed that GT1b patients with higher HCV complexity tend to have a greater chance of Y93H presence and GT3b patients are inherently resistant to current NS5A inhibitors.

## Materials and Methods

### Study Population and Sample Collection

Hepatitis C virus RNA-positive sera were collected from January to June 2018 at Guangzhou Kingmed Center for Clinical Laboratory (hereinafter referred to as Kingmed). Roughly 1/8 of total samples ordered for HCV genotyping tests were randomly selected out for NS5A RASs determination. A total of 878 unique serum samples were included in the study. Each sample was given a de-identified code. The corresponding gender and age data were recorded. HCV genotyping and subtyping were performed as described previously (Chen Y. et al., 2017).

Another set of DAA-naïve serum samples were collected from 185 GT1b-infected chronic hepatitis C patients between April 2015 and September 2017 at Department of Infectious Diseases, Ruijin Hospital, Shanghai Jiao Tong University School of Medicine (hereinafter referred to as Shanghai Ruijin). Written informed consent was obtained from all patients before the study. HCV subtype was confirmed by RT-PCR and sequencing of the HCV core region as described previously (Walker et al., 2017). No patients had ever been treated for HCV with any DAAs and fifty-two patients had documentation of experiencing interferon-based therapy at sample collection.

### Amplification and Sequencing of HCV NS5A Region

For samples collected at Kingmed, NS5A region was amplified by nested primers (Supplementary Table S1) spanning nucleotides 1–309 (6258 to 6566 nucleotides of the reference strain H77 with accession number AF009606) and the purified DNA product was sequenced by direct Sanger sequencing. Briefly, total viral RNA was extracted from 140 μL of serum with TIANamp Virus DNA/RNA Kit (Tiangen, Beijing, China) according to manufacturer’s protocol. RNA was eluted with 50 μL of nuclease-free water and kept at −80°C for future use. HCV RNA was reverse transcribed and amplified for the first round with HiScript^®^ II One Step RT-PCR kit (Vazyme, Nanjing, China) and 2 × phanta^TM^ Master Mix (Vazyme, Nanjing, China) for the second round following the manufacturer’s instructions. PCR conditions included a predenaturation step at 94°C for 3 min, followed by 35 cycles of denaturation at 94°C for 30 s, annealing at 57°C for 30 s, and extension at 72°C for 30 s. A final 5-minute extension step at 72°C was added. PCR reaction contained 25 μL of 2 × reaction premix, 1 μL of each primer (10 μM), 5 μL of cDNA template, and nuclease-free water to a final volume of 50 μL. The first round PCR product was used as the template in the second round of PCR, which was done with the same reaction volume and conditions as the first round. Sanger sequencing of PCR products from both directions was performed using an automatic sequencer (ABI Prism 3730xl, Thermo Fisher Scientific, Waltham, MA, United States). Sequencing primers were the second round PCR primers. Chromatograms were visually inspected and analyzed using Sequencher (Gene Codes Corporation, Ann Arbor, MI, United States). Mixed-calls were assigned where minority alleles composed >20% of chromatogram.

For samples collected at Shanghai Ruijin, HCV NS5A domain 1 sequence was determined by both Sanger sequencing and Next Generation Sequencing (NGS) as follows. HCV RNA was reversed transcribed and NS5A region was amplified as described previously except with the following modifications: The PCR reaction was catalyzed with Q5 High-Fidelity DNA Polymerase (New England Biolabs). For NGS multiplex sequencing, PCR products from the first round PCR reaction were amplified in the second round with nested primers which had sample-specific 7-bp barcodes additionally incorporated. PCR amplicons were purified with Agencourt AMPure Beads (Beckman Coulter, Indianapolis, IN, United States) and quantified using the PicoGreen dsDNA Assay Kit (Invitrogen, Carlsbad, CA, United States). After the individual quantification step, amplicons were pooled in equal amounts, and pair-end 2 × 300 bp sequencing was performed using the Illumina MiSeq platform with MiSeq Reagent Kit v3 at Shanghai Personal Biotechnology Co., Ltd (Shanghai, China). For the calling of nucleotide mutations and amino acid substitutions, each amplicon was compared to the reference sequence of HCV 1b-Con1 (accession number AJ238799). The technical error rate was estimated by amplifying and sequencing Con1 plasmid exactly the same as the clinical isolates. A cut-off value (1%) was set as the minimum threshold in calling the presence of polymorphism.

### Phylogenetic Analyses

The overall phylogenetic analysis was performed using 878 NS5A sequences determined from the present study and reference sequences 1b-Con1 (AJ238799), 2a-JCH-1 (AB047640), 3a-PR87 (HQ912953), 3b-HCV-Tr (D49374) and 6a-HK33 (AY859526) retrieved from GenBank. HCV NS5A 1–309 nucleotides were aligned with reference sequences. GT3a and GT3b sequences were additionally aligned with reference sequences with available information of isolation regions, including countries from Europe (France, Switzerland, United Kingdom, Italy, and Germany), Asia (China, India, Pakistan, Thailand and Japan), North America (United States), South America (Brazil) and Oceania (Australia), with details provided in the respective figure legends. The evolutionary history was inferred by using the maximum likelihood (ML) method based on the Tamura and Nei (1993). Initial tree(s) for the heuristic search were obtained automatically by applying Neighbor-Join and BioNJ algorithms to a matrix of pairwise distances estimated using the Maximum Composite Likelihood (MCL) approach, and then selecting the topology with superior log likelihood value. The tree was drawn to scale, with branch lengths measured in the number of substitutions per site. Codon positions included were 1st + 2nd + 3rd + Non-coding. All positions containing gaps and missing data were eliminated. The tree files were generated with MEGA X (Kumar et al., 2018) and visualized by interactive Tree of Life (iTOL) (Letunic and Bork, 2016).

### Characterization of NS5A Quasispecies Variants

The number and frequency of distinct virus variants in each sample were estimated using the computational method Shannon Entropy as described previously with some modifications (Margeridon-Thermet et al., 2014). Briefly, paired sequences were joined, filtered and de-replicated by USEARCH (Edgar, 2010). Singletons were removed and the remaining sequences were normalized to the same amount (3500). Then Shannon entropy was calculated as follows, Sn = −[Σ_i_ (pi×ln pi)]/ln N, in which pi is the proportion of each different sequence, and *N* is the total number (Domingo et al., 2006).

### Data Access

All 878 NS5A sequences determined by Sanger method were deposited in GenBank with accession numbers MK255325-MK256202. All NS5A NGS raw data were deposited in the NCBI Sequence Read Archive under accession numbers SAMN10458261-SAMN10458445.

### Statistical Analysis

Categorical variables were described as proportion (%), and continuous variables were described as mean ± standard error (SEM). Statistical analyses were performed using GraphPad Prism software. Comparisons among multiple groups or between two groups were performed using Chi-square test or *t*-test as appropriate. *p* < 0.05 was considered statistically significant.

## Results

### Characteristics of Study Population

The present study included 878 randomly selected unique patient samples collected at KingMed Laboratory from January to June 2018. The samples were taken from 27 provinces/municipalities (including Anhui, Beijing, Chongqing, Fujian, Gansu, Guangdong, Guangxi, Hainan, Hebei, Heilongjiang, Henan, Hubei, Hunan, Inner-Mongolia, Jiangsu, Jiangxi, Jilin, Liaoning, Qinghai, Shaanxi, Shandong, Shanghai, Shanxi, Sichuan, Tianjin, Yunnan, and Zhejiang). HCV genotyping was initially accomplished by TaqMan PCR and further validated by core-envelope 1 sequencing (Chen Y. et al., 2017). Samples were subtyped as follows: GT1b (*n* = 489), GT2a (*n* = 203), GT3a (*n* = 60), GT3b (*n* = 78) and GT6a (*n* = 48). Samples of GT1a (*n* = 1) and GT6n (*n* = 7) were excluded from the study due to the limited sample size. No samples of mixed-subtype HCV infection were detected. The general information of these patients is summarized in Table 1. GT3a, GT3b and GT6a infections are more popular in male and younger patients compared to GT1b and GT2a. The regional distribution of HCV subtypes was shown in Supplementary Figure S1. Among the 489 GT1b samples, 27.4, 33.1, 22.7 and 16.8% of them were collected from Northern, Eastern, Southern and Western China, respectively. Similarly, 53.7, 28.1, 8.4 and 9.9% of GT2a samples, 11.6, 56.5, 15.2 and 16.7% of GT3 samples were collected from Northern, Eastern, Southern and Western China, respectively. GT6a samples were only collected from Eastern (43.8%) and Southern (56.3%) China. The geographic imbalance of GT distribution is obvious. GT2a patients are more abundant in Northern China, while GT6a patients are concentrated in South-eastern China.

**Table 1:** General information of patients, stratified by Hepatitis C virus genotypes.

		GT1b	GT2a	GT3a	GT3b	GT6a
Kingmed	*n* (%)	489 (55.7)	203 (23.1)	60 (6.8)	78 (8.9)	48 (5.5)
	Age, mean ± SEM	51.6 ± 0.6	55.0 ± 0.9	43.0 ± 1.1	44.8 ± 1.2	42 ± 1.3
	Male (%)	48.7	51.2	75.9	75.3	62.5
Shanghai Ruijin	*n* (%)	185 (100)	/	/	/	/
	Age, mean ± SEM	50.4 ± 1.2	/	/	/	/
	Male (%)	42.7	/	/	/	/

### Phylogenetic Analyses of NS5A Sequences

Phylogenetic analyses of all NS5A sequences amplified were conducted to investigate the genetic relationship among them. After aligning with HCV strains of standard GTs, a 309-bp fragment of the partial NS5A region (H77 positions: 6258–6566 nucleotides) was utilized for HCV phylogenetic analyses. The phylogenetic tree included 489 GT1b sequences (North *n* = 134, East *n* = 162, South *n* = 111, West *n* = 82), 203 GT2a sequences (North *n* = 109, East *n* = 57, South *n* = 17, West *n* = 20), 60 GT3a sequences (North *n* = 7, East *n* = 31, South *n* = 11, West *n* = 11), 78 GT3b sequences (North *n* = 9, East *n* = 47, South *n* = 10, West *n* = 12), 48 GT6a sequences (East *n* = 21, South *n* = 27) and five representative reference sequences (1b-Con1, 2a-JCH-1, 3a-PR87, 3b-HCV-Tr and 6a-HK33). A circular ML phylogenetic tree is shown in Supplementary Figure S2. In the tree, sequences of the same subtype were grouped closely, and subtypes differed from each other considerably, which revealed a great diversity of HCV isolates.

**FIGURE 1:**
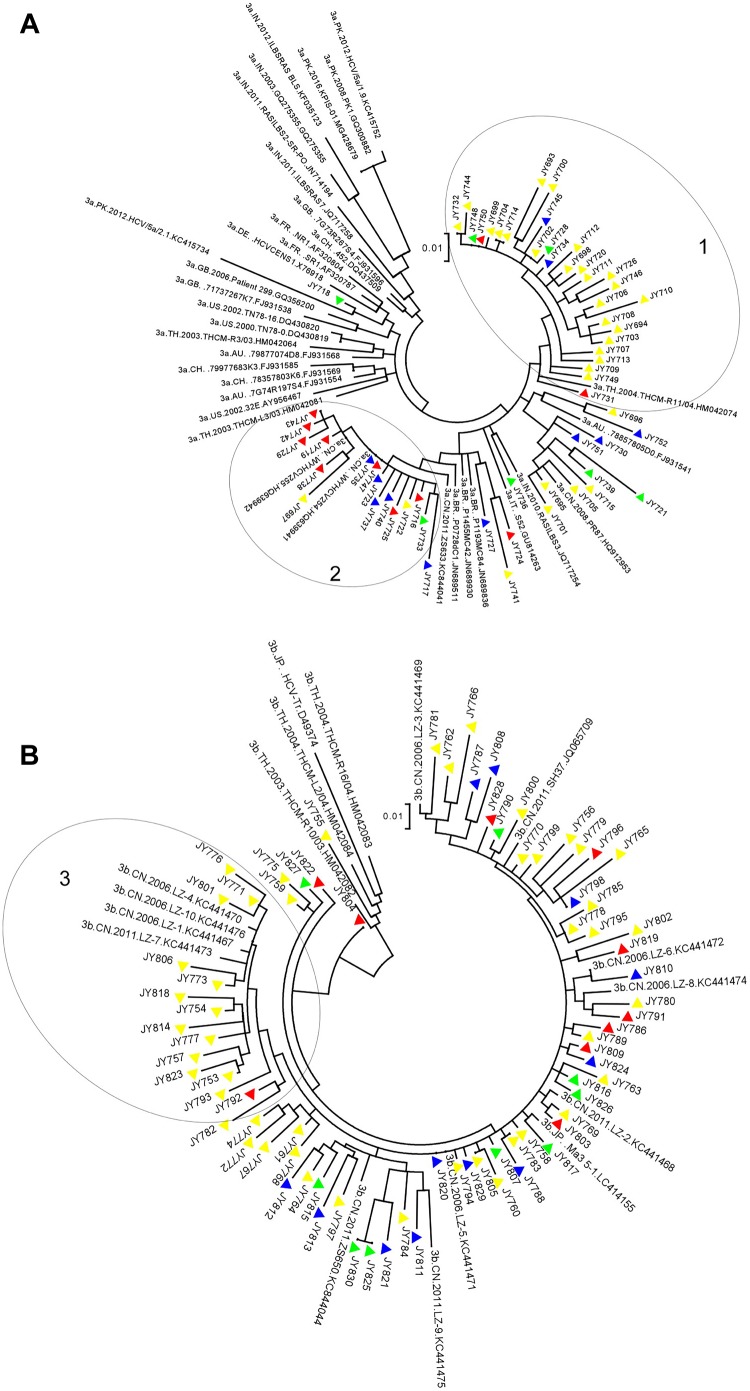
Maximum likelihood trees constructed for GT3a and GT3b NS5A sequences. The vertical scale bar represents 0.01 nucleotide substitutions per site. The triangular symbols provided next to the isolate names denote their respective collection regions: red indicates Southern China-origin, blue indicates Western China-origin, yellow indicates Eastern China-origin, and green indicates Northern China-origin, respectively. The geographic codes are: AU = Australia, BR = Brazil, CH = Switzerland, CN = China, DE = Germany, FR = France, GB = United Kingdom, JP = Japan, IN = India, IT = Italy, PK = Pakistan, TH = Thailand, US = United States. Three circles indicate cluster 1, 2 and 3. **(A)** The tree was constructed using 60 GT3a NS5A nucleotide sequences determined from this study and 35 reference sequences from China (HQ912953, KC844041, HQ639941, HQ639942), India (GQ275355, JN714194, JQ717254, JQ717258, KF035123), Pakistan (GQ300882, KC415734, MG428679, KC415752), Thailand (HM042064, HM042074, HM042081), United States (AY956467, DQ430819, DQ430820), Australia (FJ931541, FJ931554, FJ931568), Brazil (JN689511, JN689836, JN689930), France (AF320787, AF320804), Germany (X76918), Italy (GU814263), Switzerland (DQ437509, FJ931569, FJ931585) and United Kingdom (FJ931538, FJ931596, GQ356200). The tree with the highest log likelihood (–2420.88) was shown. There were a total of 263 positions in the final dataset. **(B)** The tree was constructed using 78 GT3b NS5A nucleotide sequences determined from this study and 17 reference sequences from China (JQ065709, KC441467–KC441476, KC844044), Japan (D49374, LC414155), and Thailand (HM042082, HM042083, HM042084). The tree with the highest log likelihood (–2437.25) was shown. There were a total of 267 positions in the final dataset.

Phylogenetic analyses based on HCV structure protein sequences concerning HCV isolates circulating in China have been reported previously, mainly on GT1b and GT6a sequences (Nakano et al., 2006; Fu et al., 2011, 2012; Yuan et al., 2017). The sizeable number of GT3a and GT3b NS5A sequences determined in this study gave us an opportunity to have a close look at the phylogenetic relationship between these domestic GT3 sequences and reference sequences deposited in public database. Figure 1A presents a tree of 60 GT3a sequences from this study and 35 reference sequences with different geographic origins retrieved from Los Alamos HCV database. Overall, these GT3a strains were separated from most reference isolates on the upper left corner, indicating GT3a strains circulating in China are phylogenetically distant from these references. Cluster 1 sequences were mainly from Eastern China and related to a strain isolated from Thailand in 2004 (HM042074), suggesting the origination of this cluster and its regionally epidemic distribution. Instead, cluster 2 sequences were more related to a strain isolated in 2011 from China (KC844041), and showed a mixture of geographic origins, indicating nationally epidemic distribution of this cluster. Figure 1B details a phylogenetic tree of 78 GT3b sequences from this study and 17 references sequences exclusively from Asia (2 from Japan, 3 from Thailand and 12 from China). Though sequences in cluster 3 were mainly from Eastern China, they had close genetic relationship to a few strains isolated from Liuzhou, Guangxi, China around 2006–2011 (LZ-1.KC441467, LZ-4.KC441470, LZ-7.KC441473, LZ-10.KC441476). Sequences on the right part of Figure 1B showed a scattered distribution without geographic clustering. Some isolates also had high similarity to strains collected from Liuzhou (LZ-3.KC441469, LZ-6.KC441472, LZ8-KC441474, LZ-2.KC441468, LZ5-KC441471), indicating the origination and dissemination of GT3b strains from South-western China and isolates in other regions may be viewed as descendants.

### HCV Subtype-Specific Distribution of NS5A RASs

Due to the heterogeneity of HCV sequences, the prevalence of HCV NS5A RASs was stratified by subtype. For each subtype, patient NS5A amino acid sequences were aligned with 1a-H77 isolate sequence (AF009606). Clinically relevant NS5A RASs were determined according to literatures (Kalaghatgi et al., 2016; Pawlotsky, 2016). The majority and minority variants for each subtype were reported relative to the amino acid at the indicated position. A full list of NS5A substitutions was collected (Supplementary Table S2) and the most clinically-relevant HCV RASs were highlighted in Table 2.

**Table 2:** Proportion of clinically important NS5A RASs.

subtype	RAS (Position/amino acid)	Proportion (%)	Resistance to DAAs
GT1b	28A	0 (0)	Daclatasvir
	28M	6 (1.2)	Ledipasvir, Ombitasvir
	28T	0 (0)	Ombitasvir
	28V	1 (0.2)	Ombitasvir
	31F	1 (0.2)	Elbasvir, Ombitasvir
	31I	0 (0)	Daclatasvir
	31M	10 (2.0)	Daclatasvir, Elbasvir, Ledipasvir
	31V	1 (0.2)	Daclatasvir, Ombitasvir
	32del	0 (0)	Daclatasvir, Ledipasvir, Ombitasvir
	32L	0 (0)	Daclatasvir
	58D	0 (0)	Ledipasvir
	92K	0 (0)	Daclatasvir, Ledipasvir
	93H	69 (14.1)	Daclatasvir, Elbasvir, Ledipasvir, Ombitasvir, Velpatasvir
	93N	0 (0)	Daclatasvir
	93S	1 (0.2)	Ledipasvir
GT2a	28S	0 (0)	Daclatasvir, Pibrentasvir
	31M	194 (95.6)	Daclatasvir
	31I	0 (0)	Pibrentasvir
	93H	0 (0)	Daclatasvir, Elbasvir, Ledipasvir, Ombitasvir, Velpatasvir
GT3a	30K	0 (0)	Daclatasvir, Elbasvir, Ledipasvir, Pibrentasvir, Velpatasvir
	31F	0 (0)	Daclatasvir, Elbasvir
	31M	0 (0)	Daclatasvir, Velpatasvir
	31V	0 (0)	Daclatasvir
	93H	2 (3.3)	Daclatasvir, Elbasvir, Ledipasvir, Ombitasvir, Velpatasvir
GT3b	30K	76 (97.4)	Daclatasvir, Elbasvir, Ledipasvir, Pibrentasvir, Velpatasvir
	31F	0 (0)	Daclatasvir, Elbasvir
	31M	77 (98.7)	Daclatasvir, Velpatasvir
	31V	0 (0)	Daclatasvir
	93H	0 (0)	Daclatasvir, Elbasvir, Ledipasvir, Ombitasvir, Velpatasvir
GT6a	93H	0 (0)	Daclatasvir, Elbasvir, Ledipasvir, Ombitasvir, Velpatasvir

In GT1b sequences, the proportion of Y93H was 14.1%. The residues of Q24, L28, L31, P32, S38, and A92 were highly conserved. Residues R30 and P58 were more polymorphic: 84 (17.2%) or 34 (7%) of 489 sequences harboring substitutions Q30 or S58, respectively.

In GT2a sequences, L31M was found in 95.6% of GT2a subjects, indicating a homogeneously high frequency of RAS at this amino acid. No RAS was detected at Y93 position. The proportion of T24S, F28L, K30R, P58S, and C92S was as low as 2.5, 2.5, 1.0, 1.5, and 3.0%, respectively.

Interestingly, totally different distribution patterns of RASs were noted in GT3a and GT3b isolates. Among the 60 GT3a sequences, only 3.3% were detected with Y93H and no other RASs were found. In contrast, 97.4 and 98.7% of GT3b sequences were detected with A30K and L31M mutation, respectively. Y93H was not observed in any GT3b sequences.

As for GT6a sequences, substitutions of Q24K/R (18.8%), L28F (20.8%) and T58S (6.3%) were detected, but no Y93H was found.

**FIGURE 2:**
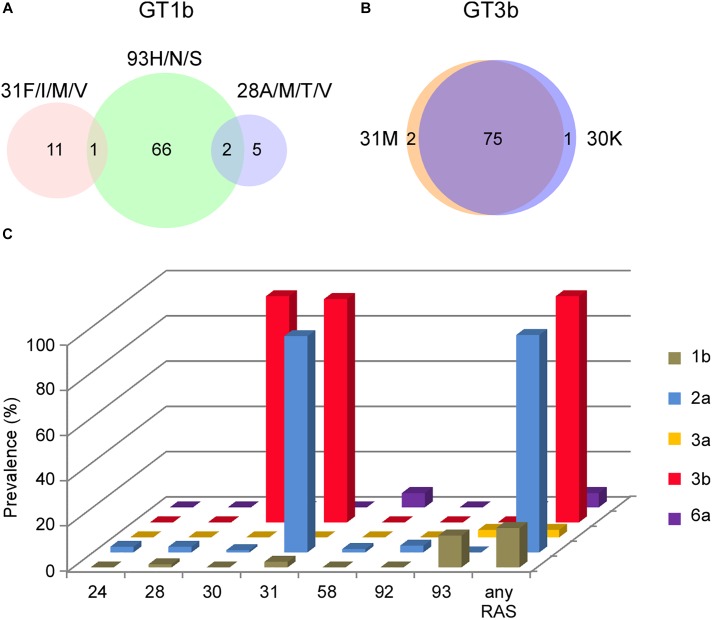
Coincidence of RASs distribution. **(A)** NS5A 28A/M/T/V, 31F/I/M/V and 93H coexistence in GT1b sequences by Venn diagram. **(B)** NS5A 30K and 31M coexistence in GT3b sequences by Venn diagram. Sample numbers were labeled in the diagram. **(C)** Prevalence of RASs detected in NS5A by subtypes. NS5A RASs definition (Wei L. et al., 2018) include: 1b: L28M/V, L31I/F/M/V, P58D, A92K and Y93C/H/N/S; 2a: T24G/N/R/S, F28A/C/G/L/T, K30E/G/H/R/T, L31I/F/M/V, P58D/S, C92K/N/S/T and Y93C/F/H/N/S; 3a or 3b: S24G/N/R, M28A/G/T, A30E/G/H/K/R, L31I/F/M/V, P58D/G, E92T and Y93C/F/H/N/S; 6a: Q24G/N, F28A/G/M/T/V, R30E/G/H/K/L/T, L31I/F/M/V, T58A/D/G/S, A92K/T and T93A/C/F/H/N/S.

Coincidence of multiple RASs may further decrease the genetic barrier to resistance and toughen the treatment selection (Gottwein et al., 2018). We analyzed the overlapping percentage of clinically important NS5A RASs among subtypes (Figure 2). In GT1b sequences, no overlapping of 28A/M/T/V + 31F/I/M/V was detected, and low frequency of 28A/M/T/V + 93H/N/S (2/489) or 31F/I/M/V + 93H/N/S (1/489) was found (Figure 2A). It’s worth noting that the coexistence of A30K + L31M was extremely high in GT3b sequences (75/78) (Figure 2B). Taken together, Y93H in GT 1b sequences, L31M in GT2a sequences, A30K and L31M in GT3b sequences, contribute most to NS5A RASs prevalence in China (Figure 2C).

### Quasispecies Diversity in GT1b Samples

Among the RASs listed, Y93H is primarily associated with reduced NS5A-targeting DAAs efficacy across all subtypes (Gottwein et al., 2018). Y93H was identified in GT1b (14.1%) but rarely seen in other subtypes. We analyzed the demographic and regional characteristic of these GT1b patients and found the age and gender distribution showed no statistical difference between patients presenting with Y93 and Y93H variants (Supplementary Table S3). When looking at the regional distribution, a significantly higher Y93H frequency was observed in the patients from Eastern area (East / non-East, 20.4% /11.0%, *p* = 0.0083).

**FIGURE 3:**
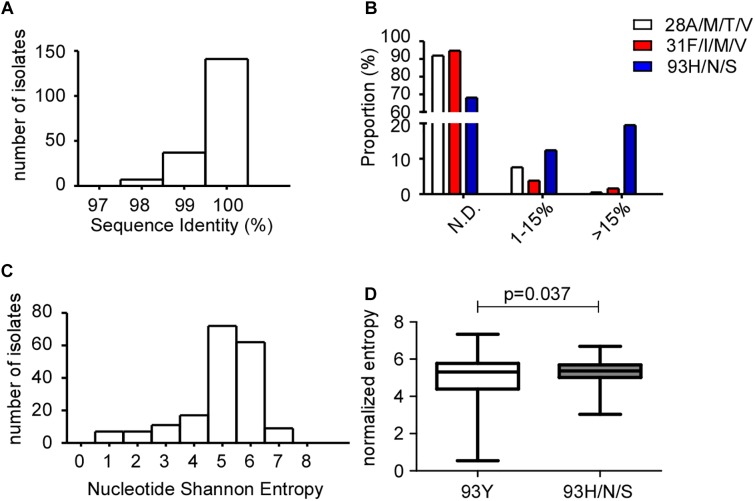
Quasispecies diversity in GT1b samples by NGS. **(A)** Baseline NS5A sequence identity between NGS and Sanger Sequencing. NGS- and Sanger-derived consensus NS5A sequences from 185 patients samples were aligned and analyzed at the nucleotide level using the BioEdit software. **(B)** Proportions of patients carrying NS5A RASs 28A/M/T/V, 31F/I/M/V or 93Y/H/S were plotted against the RASs frequency. N.D., not detected. **(C)** Histograms of baseline NS5A quasispecies diversity, as represented by Shannon entropy in nucleotides. **(D)** Box plot of normalized entropy in samples with 93Y or 93H/N/S. The unpaired *t*-test with Welch’s correction was used to assess the statistical significance of differences between the median Shannon entropy.

To rule out the sampling bias at Kingmed Laboratory and further verify the Y93H prevalence in GT1b patients, a validation set of 185 GT1b-infected DAA-naïve patients samples collected at Ruijin hospital was tested for RASs frequency (Table 1). The NS5A sequences were amplified and determined by both Sanger and NGS sequencing platforms. The sequence identity is greater than 97% at the nucleotide level for all samples (Figure 3A), indicating reliable quality of both sequencing methods. Benefiting from the increased resolving power of NGS, the cumulative frequency of Y93H/N/S is 31.9% (1% cutoff), compared to 20.5% by Sanger platform (data not shown), which is consistent with Kingmed data. Since resistant variants present in low proportions (below 15%) at baseline were deemed not to influence the response significantly (Pawlotsky, 2016), we then use 15% as threshold to report the presence of resistant variants assessed by deep sequencing. The sample proportions of 28A/M/T/V, 31F/I/M/V or 93H/N/S were plotted against variants frequencies: no mutation detected (below the 1% cut-off value), mutation frequency between 1–15%, and greater than 15% (Figure 3B). Around 12 and 19.5% samples had Y93H/N/S detected in the range of 1–15% and above 15%, respectively. The incidence of 28A/M/T/V and 31F/I/M/V is low: 7.6 or 3.8% of samples had presence of 28A/M/T/V or 31F/I/M/V with frequencies less than 15%. Only marginal number of samples had 28A/M/T/V or 31F/I/M/V detected in more than 15% of the viral population.

The quasispecies genetic diversity between GT1a and GT1b was hypothesized to contribute to a higher level of DAA resistance in GT1a patients (Margeridon-Thermet et al., 2014). To elucidate which factors lead to the Y93H presence in GT1b patients, we evaluated the intra-patient genetic variation in NS5A quasispecies on a quantitative scale. The histograms of normalized Shannon entropy of quasispecies in nucleotides were plotted for all samples (Figure 3C). The mean Shannon entropy for the NS5A nucleotide sequences was 4.959 ± 0.096. When we use 15% as cut-off value to stratify whether there is RAS at the 93rd amino acid in NS5A sequences, the mean entropy was 4.865 ± 0.117 in 93Y group, but 5.278 ± 0.126 in 93H/N/S (*p* = 0.037) (Figure 3D), which suggests patients presenting with 93H/N/S have higher HCV complexity.

## Discussion

Hepatitis C is an important cause of liver-related death. With the emergence of DAAs that have overwhelmingly transformed HCV treatment, WHO advocated the vision to eliminate the disease by 2030. However, clinical application of DAAs is highly concentrated in Europe and United States, but still rare in resource-limited countries and regions. Patients in China have less choice than those in United States or Europe, since only daclatasvir, asunaprevir, sofosbuvir, simeprevir, danoprevir, elbasvir/grazoprevir, ombitasvir/paritaprevir/ritonavir + dasabuvir (3D), sofosbuvir/ledipasvir and sofosbuvir/velpatasvir have currently been approved in mainland China for HCV treatment with different indications. For example, danoprevir can merely be applied with pegylated-interferon/ribavirin and to non-cirrhotic GT1b patients. Elbasvir/grazoprevir is suitable for GT1 and GT4 patients, while daclatasvir/asunaprevir and 3D regimen can only be applied to GT1 patients. Considering the high prices of these DAAs, reasonable and individualized treatment plans are demanded.

Benefiting from the nationwide network of sample collection by Kingmed Laboratory, we randomly selected 878 samples with different subtypes from 27 provinces/municipalities across China. Different GTs have distinct geographic and demographic distribution patterns, which are consistent with a previously published large sample size survey (Chen Y. et al., 2017). In general, GT6 isolates are more concentrated in South-eastern China while GT2 isolates are more frequently found in Northern China. GT3 and GT6 patients are younger and male dominant compared to GT1 and GT2 patients. The phylogenetic analyses of these 878 NS5A sequences confirmed a great diversity of HCV isolates. We can find both regionally and nationally epidemic clusters, as illustrated by GT3 ML trees. The separation of GT3 strains in China from strains isolated abroad indicates that the NS5A RASs distribution may be geographically different and needs surveillance.

Natural occurring NS5A RAS distribution was our initial study aim. However, due to missing information of DAA experience in Kingmed sampling procedures, we can not rule out the possibility that some of the samples may be from DAA-experienced patients. In our estimation, the ratio of DAA-experienced patients should be very low based on the following reasons. Firstly, original DAAs were not approved until the middle of 2017. For example, daclatasvir + asunaprevir was the first DAA regimen approved in mainland China in June 2017. Simeprevir, 3D regimen and sofosbuvir were then approved in China in the late 2017. Considering the treatment duration, samples collected in the first half year of 2018 would be mainly from DAA-naïve patients. Secondly, Kingmed Laboratory roughly has 14,000 samples ordered for HCV genotyping test yearly (Chen Y. et al., 2017). We randomly selected about 12% of the total samples in a 6-month time frame. The sample composition should be reflective of current situation in China, which still faces challenges in DAA application (Hu and Ren, 2016). Thus, we have good reasons to believe that most of the samples should be from DAA-naïve patients.

Given this understanding, clinically relevant NS5A RASs distribution pattern was analyzed. In GT1b patients, the most prominent RAS is Y93H. About 14% of GT1b patients had Y93H detected, which leads to a high fold change of resistance (Bagaglio et al., 2016). RASs of 28A/M/T/V or 31F/I/M/V were rarely seen. In a study conducted in 70 patients sampled from Western China, the frequency of NS5A Y93H is 7.58% (Li et al., 2017). Another large scale RASs study conducted with Chinese patient’s samples from Gilead sofosbuvir-containing clinical trials showed the frequency of NS5A Y93H in GT1b population is 15% (Wei L. et al., 2018). Interestingly, when we look at the regional distribution of Y93H in GT1b patients, we found that patients from Eastern China had a higher Y93H prevalence (Eastern/Non-Eastern regions: 20.4%/11.0%, *p* = 0.0083). To rule out biased RASs presence due to DAA experience in patients from Eastern China, which is the most populated and wealthy region and patients there may have higher chances of DAA exposure, we used 185 DAA-naïve GT1b patients samples collected at Shanghai Ruijin Hospital, harboring one of the biggest hepatitis C center located in Eastern China, to repeat the RASs study. We adopted both Sanger sequencing and deep sequencing to analyze RASs prevalence in this validation set. The detection limit of viral variants in quasispecies is usually about 10 to 25% for Sanger sequencing and 1% for NGS, respectively (Halfon and Sarrazin, 2012; Pawlotsky, 2016). Through comparing NS5A sequences determined by both methods side-by-side, we found that the Y93H prevalence is 20.5% by Sanger sequencing (20% cut off) or 19.5% by NGS sequencing (15% cut off), which is comparable to Kingmed result. This further corroborates that the results from Kingmed Laboratory reveal mainly, if not all, natural NS5A RASs prevalence pattern.

Compared to the homogeneous distribution of NS5A RASs in GT2 and GT3 isolates, the diversified distribution of Y93H in GT1b strains is intriguing. To understand the underlying reasons, we compared the NS5A sequences carrying wild-type Y93 or mutant Y93H/N/S. Using normalized Shannon Entropy as an index, we found patients with Y93H/N/S had higher entropy in NS5A sequences, which indicates a larger number of distinct sequence variants. It is understandable, since a bigger viral reservoir will have more chances to harbor drug resistant mutants. The reason why some patients have more complexed quasispecies is still under investigation. It may be related to infection duration, interferon-based therapy history, patient’s immune status or initially infected viral sequences. The association between RASs prevalence and previous exposure to pegylated-interferon/ribavirin is still under debate (Chen Z.W. et al., 2017; Caudai et al., 2018). To answer this question, studies with adequate sample size are needed. The elucidation of this phenomenon would lead to a personalized treatment decision and increased SVR rate.

Resistance associated substitutions identified in DAA-experienced patients or cell culture models gave us important clues of resistance. A European study analyzed NS5A sequences from 626 DAA-failure patients and showed Y93H was the RAS most frequently associated with failure of daclatasvir, ledipasvir, or ombitasvir in patients with GT1b infection, and L31M was associated with failure of daclatasvir or ledipasvir, but not ombitasvir (Gottwein et al., 2018). *In vitro* studies showed that variants with the pre-existing RAS of 93H acquired additional NS5A changes during escape experiments, resulting in high fitness and high resistance as well (Gottwein et al., 2018). This indicates that patients with 93H substitution will be more likely to develop other RASs and fail DAA treatment consequently. An Italian real-life study based on data from 569 interferon-free DAA-failure patients detected multiple RASs in GT1b patients. Y93H was the most frequent NS5A RAS detected at failure in both GT1b and GT3a patients, independently of the NS5A inhibitor used (Di Maio et al., 2018). Resistance data from Chinese patients are still rare due to the late approval of DAAs and limited application caused by the high cost of DAAs. Given the relatively high frequency of Y93H in Chinese GT1b population, we postulate that patients carrying baseline Y93H should definitely combine other class of DAAs, especially those of high resistance barriers to increase the chance of cure.

GT3 HCV infections are among the most difficult to treat in DAA era (Ampuero et al., 2014). Our data stress that GT3b is even more difficult to treat than GT3a. GT3b prevalence was substantially higher in China than in the North America or Europe (Wei L. et al., 2018). The RASs prevalence is low in GT3a sequences. Instead, more than 96% (75/78) GT3b sequences harbored both A30K and L31M simultaneously. NS5A A30K will cause resistance to daclatasvir, elbasvir, ledipasvir, pibrentasvir and velpatasvir, while NS5A L31M will cause resistance to daclatasvir and velpatasvir (Hernandez et al., 2013; Liu et al., 2015; Kalaghatgi et al., 2016; Lawitz et al., 2016). The combination of A30K + L31M showed resistance to pibrentasvir at low levels (>20 fold increase in EC_50_), but was highly resistant to daclatasvir, velpatasvir (both > 10,000 fold increase in EC_50_) and elbasvir (>100,000,000 fold increase in EC_50_) (Smith et al., 2018). The overlapping RASs incidence toughens the selection and may lead to treatment failure. Again, the homogeneous RAS distribution in GT3b patients argues against the effects caused by DAA experience. Currently global epidemiology studies did not differentiate GT3a and GT3b infections, and most clinical trials or RASs prevalence studies were conducted in GT3a instead of GT3b patients (Gower et al., 2014; Messina et al., 2015; Fathi et al., 2017; Smith et al., 2018; Wei B. et al., 2018). In our opinion, GT3b should be studied separately and more thoroughly considering its natural resistance to NS5A inhibitors.

## Conclusion

In conclusion, the prevalence of NS5A RASs can be frequent and diversified depending on HCV genotype/subtype and treatment regimen. Y93H in GT 1b, L31M in GT2a, A30K and L31M in GT3b NS5A sequences, contribute most to NS5A RASs prevalence in China. DAA-naïve GT1b patients with greater viral complexity had higher prevalence of NS5A 93H substitution. GT3b patients are naturally resistant to NS5A inhibitors. The information of baseline NS5A RASs may provide clinical treatment guidance. Due to the essential role of NS5A inhibitors, high cross resistance among different NS5A inhibitors, and long-term persistence of NS5A inhibitor-resistant HCV variants, patients who have baseline NS5A RASs should be cautious in their DAAs selection.

## Ethics Statement

The data collection and analysis for publication was approved by the Human Ethics Committee of Ruijin Hospital, Shanghai Jiao Tong University School of Medicine.

## Author Contributions

JL, YF, LC, XG, XL, WT, and QX designed the research, analyzed results and drafted the manuscript. JL, YF, ZZ, XL, WC, HW, HZ, XG, and QX contributed to the clinical data collection and interpretation. All authors read and approved the submitted version.

## Conflict of Interest Statement

The authors declare that the research was conducted in the absence of any commercial or financial relationships that could be construed as a potential conflict of interest.

## Footnotes

1 https://hcv.lanl.gov
