# Supplementary material for: Subtype-Specific Prevalence of Hepatitis C Virus NS5A Resistance Associated Substitutions in Mainland China
Source: Front Microbiol. 2019 Mar 19;10:535. doi: 10.3389/fmicb.2019.00535 (PMC6433824; doi:10.3389/fmicb.2019.00535)
Supplement: Supplementary file 1 [file Data_Sheet_1.PDF]

Supplementary Materials

Supplementary Tables

Table S1. Primers used in this study.

Primer Name	Primer Sequence
GT1b.5AF1	ACTATGTGCCTGAGAGCGA
GT1b.5AR1	TGGTCATGCCCGTCACGTA
GT1b.5AF2	CTCTCCAGCCTTACCATCAC
GT1b.5AR2	GTAACCTCCACGTACTCCTC
GT2a.5AF1	TCATGTCTGGCGAGAAGC
GT2a.5AR1	CCCAGGAAAAGAACTCTGGAGA
GT2a.5AF2	AAGGYGCGGTCCAATGGATG
GT2a.5AR2	GTCACCTCCGCGTACTC
GT3.5AF1	GGTRCAGTGGATGAACAG
GT3.5AR1	CTGTCGAAGGTAACCTTCTTCTG
GT3.5AF2	CAGTGGATCAAYGARGACTA
GT3.5AR2	AGGRTCTCKCAACATCGAGGT
GT6a.5AF1	GCYTTYAAGATCATGAGYGG
GT6a.5AR1	GGCTCNGGYTCRCANGG
GT6a.5AF2	GTCGTGTGTGCTGCCATCTTA
GT6a.5AR2	GCACCTGGCAAGGGCACTT

Table S2. Proportion of NS5A substitutions.

Subtype	Patients (n)	NS5A amino acid substitution proportion (%) [a,b,]								
		K24	M28	Q30	L31	P32	S38	H58	A92	Y93
1b	489	Q (99.2)<br>K (0.4)<br>R (0.4)	L (99.2)<br>M (1.2)<br>V (0.2)	R (85.5)<br>Q (17.2)<br>K (0.2)<br>M (0.2)	L (98.8)<br>M (2.0)<br>F (0.2)<br>V (0.2)	-	-	P (92.2)<br>S (7.0)<br>T (1.2)<br>A (0.6)<br>L (0.6)<br>R (0.4)	A (98.4)<br>T (1.4)<br>V (1.4)<br>E (0.4)	Y (94.1)<br>H (14.1)<br>P (0.2)<br>S (0.2)
2a	203	T (91.1)<br>A (8.9)<br>S (2.5)	F (97.5)<br>L (2.5)<br>V (0.5)	K (99.5)<br>R (1.0)	M (95.6)<br>L (4.4)	-	-	P (98.5)<br>S (1.5)	C (97.5)<br>S (3.0)	-
3a	60	S (98.3)<br>A (1.7)	-	A (96.7)<br>M (1.7)<br>S (1.7)<br>T (1.7)<br>V (1.7)	-	-	-	P (100.0)	E (100.0)	Y (98.3)<br>H (3.3)
3b	78	S (98.7)<br>A (1.3)	-	K (97.4)<br>R (2.6)	M (98.7)<br>L (1.3)	-	-	P (100.0)	E (100.0)	-
6a	48	Q (87.5)<br>K (14.6)<br>R (4.2)	L (81.3)<br>F (20.8)	R (100.0)	-	-	-	T (95.8)<br>S (6.3)	-	T (100.0)

[a] Substitution proportion was evaluated at ≥20% cutoffs by Sanger sequencing.

[b] Dash (-) indicates no change from GT1a H77 reference sequence and no minority substitutions detected in analysis.

Table S3. Demographic and geographic characteristics and GT1b patients from Kingmed Laboratory.

	Y93	Y93H	*p* value
Number of patients, n (%)	420 (85.9%)	69 (14.1%)	
Mean age (years)	52.0±0.7	49.5±1.9	0.2782
Gender†, M/F	200/31	207/36	0.6939
Regions			0.0419
North, n (%)	120 (89.6)	14 (10.4)	
East, n (%)	129 (79.6)	33 (20.4)	
West, n (%)	74 (90.2)	8 (9.8)	
South, n (%)	97 (87.4)	14 (12.6)	
Regions			0.0083
East, n (%)	129 (79.6)	33 (20.4)	
Non-East, n (%)	291 (89.0)	36 (11.0)	

†Data of gender were missing for 13 patients with Y93 and 2 patients with Y93H.

Supplementary Figures

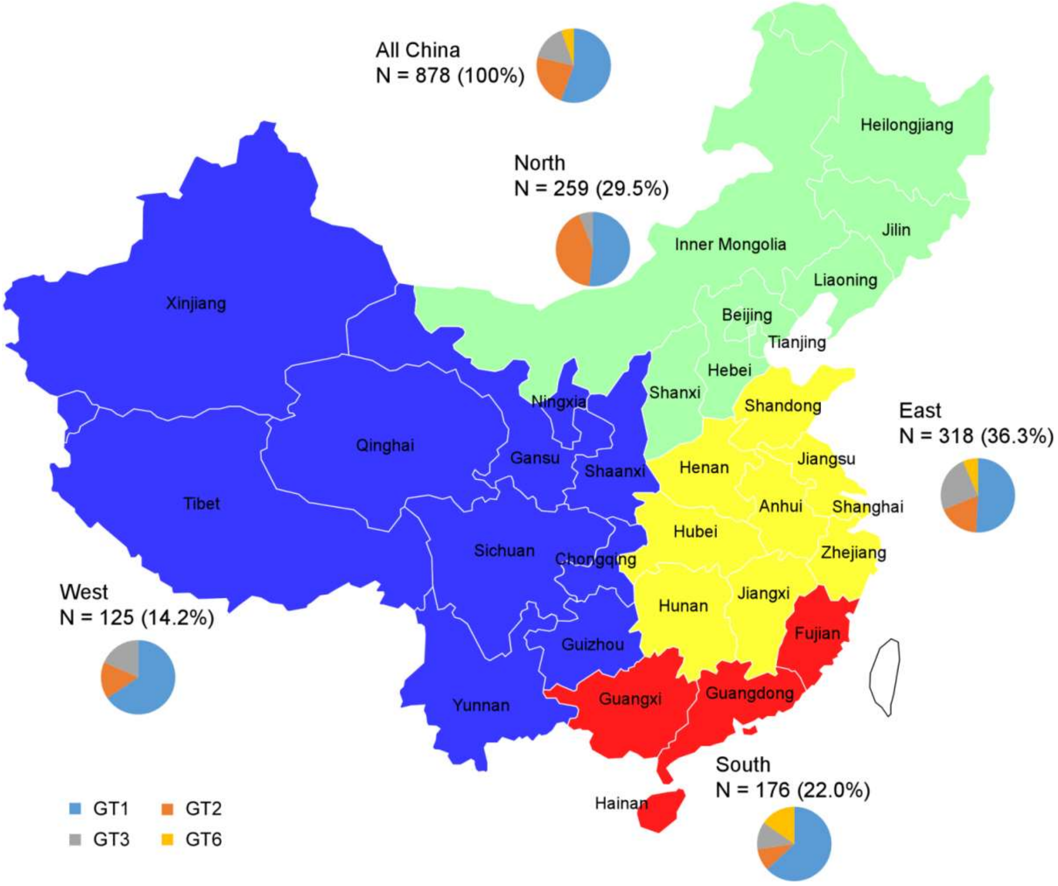

Figure S1. Distribution of patient samples stratified by genotypes and regions.

Regions defined in this study are color-coded as follows: blue-colored Western China, including Chongqing, Gansu, Guizhou, Ningxia, Qinghai, Shannxi, Sichuan, Tibet, Xinjiang, and Yunnan; red-colored Southern China, including Fujian, Guangdong, Guangxi, and Hainan; yellow-colored Eastern China, including Anhui, Henan, Hubei, Hunan, Jiangsu, Jiangxi, Shandong, Shanghai and Zhejiang; green-colored Northern China, including Beijing, Hebei, Heilongjiang, Inner-Mongolia, Jilin, Liaoning, Shanxi, and Tianjin. The distribution of subtypes in a given region is shown in Pie Chart.

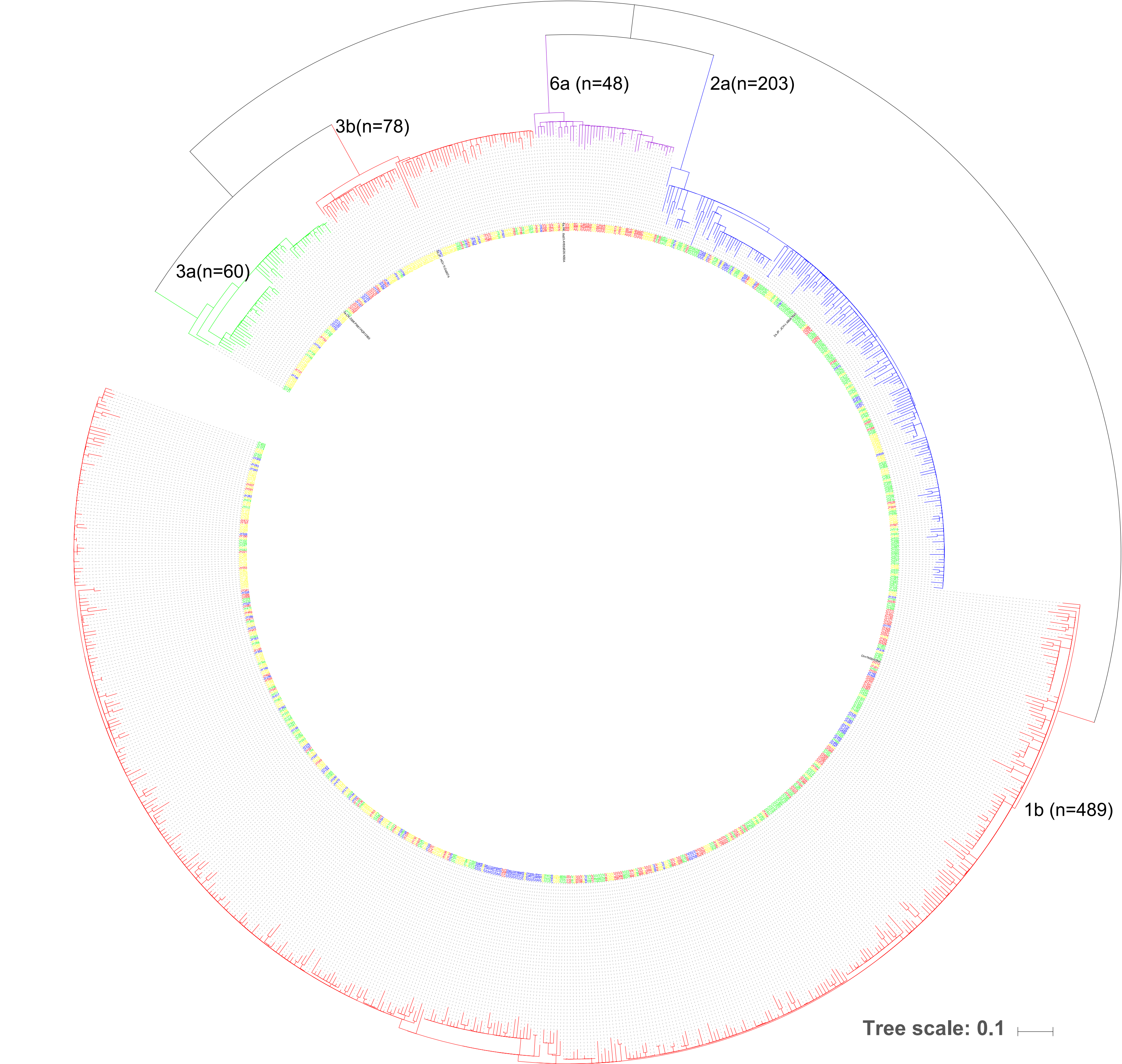
6a (n=48)
2a(n=203)
3b(n=78)
3a(n=60)
1b (n=489)
Tree scale: 0.1

Figure S2. **Phylogeny of NS5A sequences.** Circular phylogenetic tree based on partial NS5A sequences determined from 878 Kingmed samples and 5 reference sequences retrieved from GenBank, including 1b-Con1 (accession number AJ238799), 2a-JCH-1 (AB047640), 3a-PR87 (HQ912953), 3b-HCV-Tr (D49374) and 6a-HK33 (AY859526). The horizontal scale bar represents 0.1 nucleotide substitutions per site. JY001-489, JY490-692, JY693-752, JY753-830 and JY831-878 were of GT1b, GT2a, GT3a, GT3b and GT6a infections, respectively. Sample names are colored according to their geographic origins: red indicates Southern China-origin, blue indicates Western China-origin, yellow indicates Eastern China-origin, and green indicates Northern China-origin, respectively. Names of reference sequences were in black.
